# Therapeutic Potential of TNFα and IL1β Blockade for CRS/ICANS in CAR-T Therapy *via* Ameliorating Endothelial Activation

**DOI:** 10.3389/fimmu.2021.623610

**Published:** 2021-05-19

**Authors:** Yunshuo Chen, Ranran Li, Siqi Shang, Xuejiao Yang, Lei Li, Wenbo Wang, Yueying Wang

**Affiliations:** ^1^Shanghai Institute of Hematology, State Key Laboratory of Medical Genomics, National Research Center for Translational Medicine at Shanghai, Rui Jin Hospital, Shanghai Jiao Tong University School of Medicine, Shanghai, China; ^2^Department of Critical Care Medicine, Rui Jin Hospital, Shanghai Jiao Tong University School of Medicine, Shanghai, China

**Keywords:** coagulation, leakage, inflammatory response, cytokine release, CAR-T immunotherapy

## Abstract

Severe cytokine release syndrome (CRS) and immune effector cell-associated neurotoxicity syndrome (ICANS) strongly hampered the broad clinical applicability of chimeric antigen receptor T cell (CAR-T) therapy. Vascular endothelial activation has been suggested to contribute to the development of CRS and ICANS after CAR-T therapy. However, therapeutic strategies targeting endothelial dysfunction during CAR-T therapy have not been well studied yet. Here, we found that tumor necrosis factor α (TNFα) produced by CAR-T cells upon tumor recognition and interleukin 1β (IL1β) secreted by activated myeloid cells were the main cytokines in inducing endothelial activation. Therefore, we investigated the potential effectiveness of TNFα and IL1β signaling blockade on endothelial activation in CAR-T therapy. The blockade of TNFα and IL1β with adalimumab and anti-IL1β antibody respectively, as well as the application of focal adhesion kinase (FAK) inhibitor, effectively ameliorated endothelial activation induced by CAR-T, tumor cells, and myeloid cells. Moreover, adalimumab and anti-IL1β antibody exerted synergistic effect on the prevention of endothelial activation induced by CAR-T, tumor cells, and myeloid cells. Our results indicate that TNFα and IL1β blockade might have therapeutic potential for the treatment of CAR-T therapy-associated CRS and neurotoxicity.

## Introduction

Chimeric antigen receptor T cell (CAR-T) therapy following lymphodepletion has shown unprecedented prospect in the field of tumor treatment, especially in hematological malignancies. Three human B cell antigen CD19-targeted CAR-T products, Tisagenlecleucel (Kymriah), Axicabtagene Ciloleucel (Yescarta), and Lisocabtagene maraleucel (liso-cel) have been approved by Food and Drug Administration (FDA) for the treatment of relapse/refractory acute B lymphocytic and diffuse large B cell lymphoma (1–4). In the clinical trials of CAR-T therapy, the complete remission rate of pediatric/adult patients with relapsed/refractory B-cell acute lymphoblastic leukemia (B-ALL) and adult patients with relapsed/refractory B-cell non-Hodgkin lymphomas (B-NHL) is about 70%~90% and 43%~54%, respectively (5–7). However, the subsequent severe toxicities remain huge roadblocks and clinical challenges for the wide application of CAR-T therapy in the clinic (3, 8). Cytokine release syndrome (CRS) is the most common acute adverse effects of CAR-T therapy (9). Studies have shown that CRS of any grade occurred in 35%~93% of patients with B-cell lymphoma and about 80% of patients with B-ALL during CAR-T treatment respectively, which can lead to multiple organ dysfunction and even death in severe cases (10–12). Immune effector cell-associated neurotoxicity syndrome (ICANS), which commonly characterized by headache, delirium, seizures, coma, cerebral edema, and motor deficits, is another common complication of CAR-T therapy (13). Although current treatments effectively abated the severity of CRS and substantially extended event-free survival (14, 15), there are still patients unresolved, particularly in the most severe cases. This seriously limited the efficacy and the clinical applicability of CAR-T therapy. Therefore, understanding the mechanisms of CAR-T-induced CRS and ICANS is urgent for effective identification and intervention.

The occurrence of CRS is a complex network system involved by a series of cells. In summary, CAR-T cells are highly activated and proliferated after engaging to tumor-associated antigens in a short period of time. Activated CAR-T cells kill tumor cells, and at the same time they interact with bystander cells (monocytes, macrophage, antigen presenting cells, endothelial cells and so on) to activate the immune system to further exacerbate the imbalance of pro-inflammatory and anti-inflammatory systems, causing the release of a series of cytokines to result in the occurrence of CRS-related symptoms (11, 13). The mechanisms of CRS have been extensively explored, whereas the mechanisms underlying ICANS remain still obscure.

Several studies have revealed that the occurrence of CRS and ICANS could be mediated by myeloid cells (14, 15). Recently, evidences have revealed that the occurrence of CRS and ICANS is closely related to the activation of vascular endothelial cells. Patients with CRS and ICANS have shown clinical evidences of strong endothelial activation, including inflammation, coagulopathy, and enhanced vascular permeability (12, 16, 17). Moreover, patients with activated endothelial cells prior to CAR-T cell infusion were more likely to develop CRS and ICANS (12).

Although the pivotal role of endothelial cells in CAR-T therapy-associated CRS and ICANS has been recognized, the mechanisms of CAR-T therapy-induced endothelial dysfunction as well as potential therapeutic strategies have not been well studied yet. Our present study aimed to investigate the effects of the main cytokines derived from CAR-T cells and myeloid cells on endothelial activation as well as the underlying mechanisms, therefore to shed light on potential therapeutic strategies for CAR-T therapy-associated toxicities.

## Materials and Methods

### Cells and Reagents

Nalm6 (a cell line of B cell acute lymphoblastic leukemia, ATCC^®^ CRL­3273™) and HEK-293T cell line (ATCC^®^ ACS4500™) were obtained from ATCC. Nalm6 cells were transduced with a luciferase-ZsGreen lentivirus (Hanbio Biotechnology, China) and the purity of 95~100% was used for subsequent experiments.

Primary human peripheral blood mononuclear cells (PBMC) were obtained from MT-BIO (PB010C). Thawed PBMC were cultured in PRIME-XV T cell CDM (91154, Irvine SCIENTIFIC) supplemented with 100 IU/ml human recombination IL2. Primary human umbilical vein endothelial cells (HUVEC) were purchased from Lonza (CC2519, Basel, Switzerland) and grown in endothelial cell growth medium (EGM2, Lonza) supplemented with 5% fetal bovine serum (FBS) and 100 U/ml streptomycin/penicillin. HUVEC at passage 1 to 5 were used for all experiments.

For intervention experiments, adalimumab (502922, BioLegend), anti-hIL1β-IgG (mabg-hi1β-3, BioLegend), mouse control IgG1 (mabg-ctrlm, Invitrogen), and PF-562271 (S7357, Selleck) were supplemented in co-cultured supernatants as indicated.

### Preparation of Co-Cultured Supernatants

The human CD19 (hCD19) CAR-T cells were co-cultured with Nalm6 at different E:T ratios of 0:1, 1:1, 2:1, 10:1 overnight and supernatants were harvested as sCAR-T. The hCD19 CAR-T cells and Nalm6 were co-cultured with PBMC at a ratio of hCD19 CAR-T:Nalm6:PBMC = 2:1:2 overnight and supernatants were harvested as sCAR-T/PBMC. The supernatants were centrifuged and strained with 0.45 µm filter before use. Endothelial cells incubated with the supernatant of CAR-T cultured alone was taken as the negative control.

### Real-Time Quantitative Polymerase Chain Reaction

Total RNA was extracted from cell lysates in TRIzol^®^ Reagent (15596-026, Thermo Fisher Scientific). Reverse transcription of RNA was carried out using the RevertAid First Stand cDNA Synthesis Kit (K1622, Thermo Fisher Scientific). SYBR Green reagent (A25777, Thermo Fisher Scientific) was used for qRT-PCR. The detailed sequences of primers were listed in **Supplementary Table 1**.

### RNA Sequencing

Total RNA was extracted from endothelial cells stimulated with sCAR-T or TNFα. RNA purified using Ribo-Zero rRNA Removal Kit. The concentration and purity of isolated RNA were measured with ND-800 spectrophotometer. RNAs libraries were constructed with Truseq™ RNA Library prep Kit. Sequencing was carried out using a 2 x 150 bp PE configuration. The clean data (reads) were mapped using Hisat2 (version 2.1.0). Then, we performed gene expression analysis using RSEM (version 1.3.1). Differential expression analysis among different groups were analyzed using DESeq2, and then |FC| > 2 and FDR < 0.05 were determined as thresholds to different expression genes (DEGs). DEGs were validated by RT-PCR. Gene Ontology (GO) analysis was used to calculate the most significant biological process of a particular gene set (q < 0.05) according to the filtered DEGs. Venn analysis was used to calculate the number of genes/transcripts in each gene set and the overlap relationship between different gene sets. Biological Pathway enrichment analysis was based on KEGG pathway database. KEGG terms with q < 0.05 were considered to be significantly enriched.

### Flow Cytometry

Cells were harvested and stained using antibodies for the cell surface markers E-selectin VCAM1, and ICAM1 (551111, 551146, and 551144, BD). The mixture was incubated in 100 μl PBS supplemented with 2% FBS in darkness for 45~60 min at 4°C before detection. An isotype-matched antibody served as a negative control. Flow cytometry was performed on eight-laser cytometer (BD). Data were analyzed using FlowJo software.

### Western Blot

Protein samples were analyzed as previously described (18). Antibodies against phosphorylated p38 (4511), p38 (8690), phosphorylated ERK1/2 (4370), ERK1/2 (4695), phosphorylated JNK (4668), JNK (9258), phosphorylated FAK (Tyr397, 8556), FAK (3285), IκB (4814), and GAPDH (8884), anti-mouse secondary antibody (7076) and anti-rabbit secondary antibody (7074) were purchased from Cell Signaling Technology (Danvers, MA). Antibody against TF (sc-393657) was purchased from Santa Cruz. Images were taken using Amersham Imager 600.

### ELISA

The concentrations of IL6 (430504, BioLegend), IL8 (431504, BioLegend), and Angiopoietin 2 (CT527A, U-CyTech biosciences) in supernatants were measured by ELISA according to manufacturer’s instructions.

### Measurement of Cytokines and Chemokines

The supernatants were analyzed by Luminex X-200 milliplex with a 15-plex human cytokine panel (LXSAHM-15, R&D system).The kit included the following targets: GM-CSF, IFNγ, IL1β, IL1RA, IL2Rα, IL6, IL8, IL10, IL15, MCP-1,TNFα, VEGF-A, vWF-A2, IL6Rα, and PAI-1. Briefly, a mix of the 15 factor standards was serially diluted and assayed to generate standard curves for each factor. Standard curves were plotted as Log2[cytokine] vs Log2 median fluorescence/bead and a regression line was fitted to each cytokine assay for each experiment.

For the measurement of cytokines and chemokines in sCAR-T/Nalm6/PBMC, a beads-based LEGENDplexTM multi-analyte flow assay ([IL1β, IFNα2, IFNγ, TNFα, MCP-1, IL-6, IL8, IL10, IL12p70, IL17A, IL18, IL23 and IL33], 13-plex Human Inflammation Panel 1, 740809, BioLegend) was used according to the manufacturer’s protocol and detected on eight-laser Flow Cytometers (BD). Data were analyzed *via* Legendplex Version 8.

### Endothelial Barrier Permeability Assay

Permeability was quantified by measuring the flux of Evans blue-bound bovine serum albumin (BSA). Briefly, HUVEC were seeded on 6.5 mm-diameter Transwell (4 µm pore size polycarbonate filter) at 1.2 × 10^5^ cells/well and cultured to confluence. On stimulation, medium in the upper chambers was replaced with indicated medium. After stimulation, the medium in the upper chamber was replaced with 0.67 mg/ml Evans blue diluted in medium containing 4% BSA. After 30 minutes of incubation, the optical density of samples from the lower chambers was measured using a spectrophotometer at 620 nm.

### Statistical Analysis

The data were expressed as the Mean ± SD of three independent experiments. All multiple groups were compared with ordinary one-way analysis of variance (ANOVA) on Ranks followed by Bonferroni’s multiple comparisons test. All statistical analyses were performed with GraphPad Prism 8.0. * represents p < 0.05, ** represents p < 0.01, *** represents p < 0.001, and **** represents p < 0.0001.

The details of hCD19-CAR lentivirus generation, CAR-T cell manufacture, cytotoxicity assay, and small interfering RNA-mediated gene silencing were described in **Supplementary Materials**.

### Data Availability

Raw sequencing data has been deposited in the National Omics Data Encyclopedia (NODE) (http://www.biosino.org/node/project/detail/OEP001728).

## Results

### Engaged CAR-T Cells-Derived Cytokines Strongly Activated Endothelial Cells

**Figure 1:**
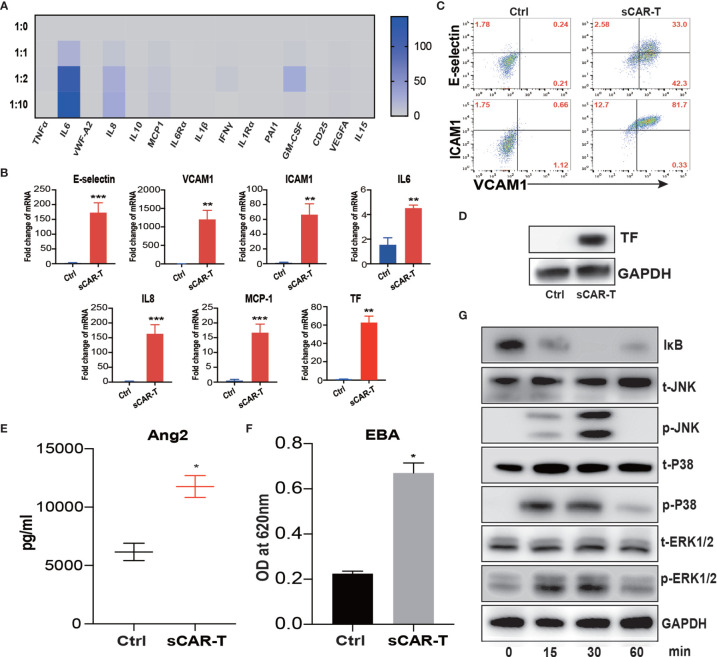
Supernatant of CAR-T/Nalm6 co-culture induced endothelial activation. **(A)** The cytokine release profile in the supernatant of HUVEC incubated with sCAR-T was determined by Luminex. The Y axis represented Target to Effector ratio. **(B)** The mRNA levels of endothelial activation-associated markers were determined by RT-PCR. GAPDH was taken as the housekeeping gene and data was expressed as fold changes relative to control. n = 3. **(C)** The protein expression of E-selectin, VCAM1, and ICAM1 in HUVEC induced by sCAR-T incubation was determined by flow cytometry. **(D)** The protein level of tissue factor (TF) in HUVEC induced by sCAR-T incubation was analyzed by western blot. **(E)** The concentration of Ang2 secreted by HUVEC was assessed by ELISA. n = 3. **(F)** The permeability of endothelial monolayer was determined by Evans blue/BSA assay (EBA). n = 3. **(G)** The protein levels of indicated proteins were determined by western blot. * represents p < 0.05, ** represents p < 0.01, and *** represents p < 0.001. ns represents not significant. All data were representative of at least three independent experiments.

To investigate the effects of activated CAR-T cells on endothelial cells, we stimulated HUVEC with the supernatants of anti-CD19 CAR-T cells/Nalm6-luc co-culture at different T:E ratios (1:0, 1:1, 1:2, 1:10) overnight. The supernatants of stimulated HUVEC were analyzed for cytokine profiling. As shown in **Figure 1A**, the levels of IL6 and IL8 were highly increased in the supernatant of HUVEC in a T:E ratio-dependent manner. The protein levels of IL6 and IL8 in the Luminex results were verified by ELISA as shown in **Supplementary Figure 1**. The absolute levels of different cytokines and factors were shown in **Supplementary Table 2**. The T:E ratio at 1:2 was selected for subsequent experiments.

Moreover, our results showed that pro-inflammatory molecules including E-selectin, VCAM1, ICAM1, IL6, IL8, and MCP-1, as well as pro-coagulant molecule tissue factor (TF) were all significantly upregulated both at mRNA and protein levels upon the incubation of sCAR-T (**Figures 1B–D**). Moreover, the level of endothelial integrity-related marker Angiopoietin 2 (Ang2) was significantly increased (**Figure 1E**). By assessing endothelial barrier function, we found that sCAR-T incubation significantly increased the permeability of HUVEC monolayer (**Figure 1F**).

Mitogen-activated protein kinases (MAPKs) and NF-кB pathway play critical roles in regulating endothelial activation. Our results showed that sCAR-T induced the phosphorylation of ERK1/2, p38, and JNK as well as the degradation of IкB (**Figure 1G**). These data suggest that MAPK and NF-кB signaling participated in sCAR-T-induced endothelial activation.

### TNFα Released by Engaged CAR-T Cells Was the Main Mediator in Inducing Endothelial Activation

**Figure 2:**
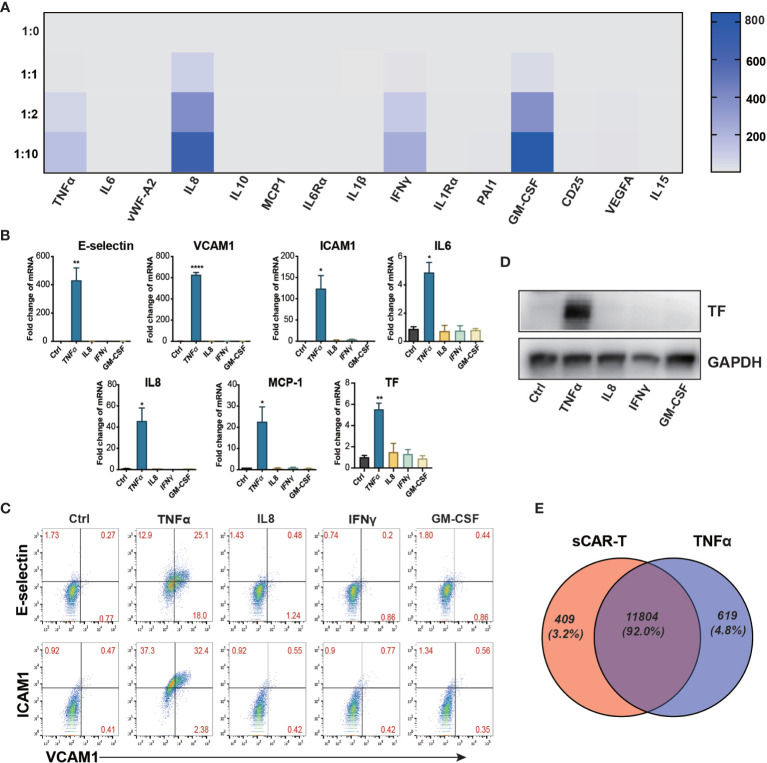
TNFα was the main mediator released by engaged CAR-T cells in inducing endothelial pro-inflammatory response. **(A)** The cytokine in the co-cultured supernatant of CAR-T/Nalm6 (sCAR-T) was analyzed by Luminex. **(B)** HUVEC were stimulated with TNFα (10 μg/ml), IFNγ (50 μg/ml), IL8 (25 μg/ml), and GM-CSF (50 μg/ml) respectively for 4h. The mRNA expression of endothelial activation-related markers was determined by quantitative RT-PCR. GAPDH was taken as the housekeeping gene and data was expressed as fold changes relative to control. **(C)** The protein expression of adhesion molecules E-selectin, VCAM1, and ICAM1 was determined by flow cytometry. **(D)** The protein expression of TF in HUVEC was determined by western blot. **(E)** The venn analysis of HUVEC stimulated with sCAR-T and TNFα. * represents p < 0.05, ** represents p < 0.01, and **** represents p < 0.0001. ns represents not significant. All data were representative of at least three independent experiments.

sCAR-T was analyzed to explore the potential cytokines contributing to the pro-inflammatory effect of engaged CAR-T cells on endothelial cells. The results showed that TNFα, IL8, IFNγ, and GM-CSF were the most abundant pro-inflammatory cytokines secreted by CAR-T cells upon tumor engagement in a T:E ratio-dependent manner (**Figure 2A**). The absolute levels of different cytokines and factors were provided in **Supplementary Table 3**. To evaluate the effects of each main cytokine mentioned above on endothelial activation, we stimulated HUVEC with human recombinant TNFα, IL8, IFNγ, and GM-CSF respectively. Interestingly, our results showed that TNFα highly activated endothelial cells by upregulating the expression of adhesion molecules (E-selectin, VCAM1, ICAM1) and cytokines (IL6, IL8, MCP-1) as well as TF, whereas IL8, IFNγ, and GM-CSF did not show any inductive effect (**Figures 2B–D**). Therefore, we speculated that TNFα is the key cytokine that contributes to the pro-inflammatory effect of engaged CAR-T cells on endothelial cells. To confirm this, we compared the transcriptome profile of HUVEC stimulated with sCAR-T and HUVEC stimulated with TNFα, and found a significant overlapping (about 91%) of the DEGs between these two groups (**Figure 2E**). The detailed overlapping genes information was shown in **Supplementary Table 4**. These results suggest that TNFα is the main mediator of engaged CAR-T-induced endothelial activation.

### RNA Expression Profiling of sCAR-T-Activated Endothelial Cells

**Figure 3:**
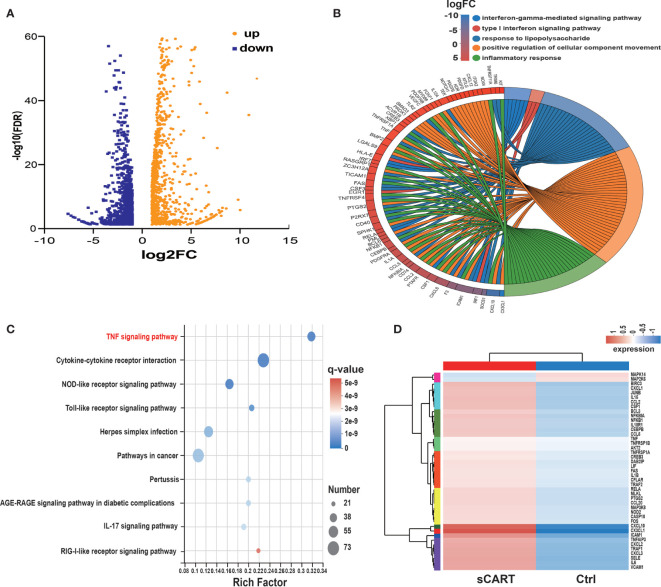
RNA expression profiling of sCAR-T-activated endothelial cells. **(A)** Differentially expressed genes (985 up-regulated and 1140 down-regulated) were shown in the Volcano Plot. **(B)** GO term enrichment analysis of differentially expressed genes. **(C)** The top ten significantly enriched pathways were obtained by KEGG analysis. **(D)** TNF signaling-associated genes were shown in the heatmap.

Comprehensive transcriptome profiling of HUVEC with or without sCAR-T incubation was analyzed by RNA-seq technique. The differentially expressed genes (DEGs) were mapped and visualized in heatmap based on R program. The results showed that among the 2125 DEGs, 985 genes were significantly upregulated and 1140 genes were downregulated in sCAR-T-stimulated HUVEC compared to control (**Figure 3A**, **Supplementary Table 5**). We examined the mRNA expression of the key genes involved in endothelial activation using quantitative real-time PCR and found that the expression levels of selected genes were in consistent with the RNA-seq results (**Supplementary Figure 2**). By assessing the involved biological process of these DEGs (genes with |FC| >2 and FDR <0.05) using the Gene Ontology (GO) approach, we found that CART-induced DEGs in HVUEC were mainly enriched in interferon-gamma-mediated signaling pathway, type I interferon signaling pathway, and inflammatory response (**Figure 3B**, **Supplementary Table 6**). In addition, Koto Encyclopedia of Genes and Genomes (KEGG) pathway analysis demonstrated that these DEGs are primarily mediated by TNF signaling pathway, cytokine-cytokine receptor interaction, NOD-like receptor signaling pathway, as well as Toll-like receptor signaling (**Figure 3C**). The heatmap (**Figure 3D**) showed the changes of TNFα signaling-associated genes induced by sCAR-T incubation. These data provided us an overview of the transcriptional regulation of endothelial activation induced by engaged CAR-T cells.

### Neutralization of TNFα Ameliorated Endothelial Activation Without Impairing the Effector Functions of CAR-T Cells

**Figure 4:**
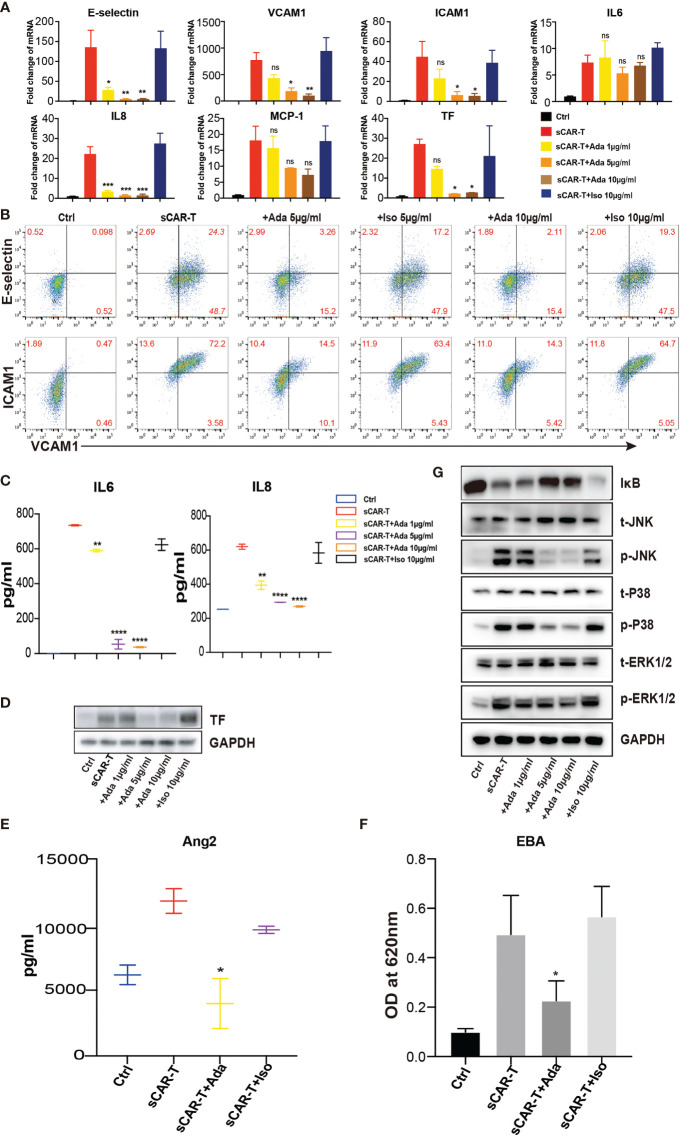
The effects of adalimumab on endothelial activation induced by sCAR-T. HUVEC were incubated with sCAR-T supplemented with different doses of adalimumab (1 µg/ml, 5 µg/ml, and 10 µg/ml) or isotype control for 4h. **(A)** The mRNA expression of endothelial activation-related markers was determined by quantitative RT-PCR. **(B)** The protein expression of E-selectin, VCAM1, and ICAM1 was determined by flow cytometry. **(C)** The concentration of IL6 and IL8 in the supernatant was determined by ELISA. **(D)** The protein expression of TF in HUVEC was determined by western blot. **(E)** The concentration of Ang2 secreted by HUVEC was assessed by ELISA. n = 3. **(F)** Confluent HUVEC cultured in Transwell were incubated with sCAR-T supplemented with or without adalimumab for 12h. The permeability of endothelial monolayer was determined by EBA. **(G)** The protein levels of indicated proteins were analyzed by western blot. * represents p < 0.05, ** represents p < 0.01, *** represents p < 0.001, and **** represents p < 0.0001. ns represents not significant. All data were representative of at least three independent experiments.

In order to investigate the effects of TNFα blockade on endothelial activation, HUVEC were incubated with sCAR-T in the presence of TNFα neutralizing antibody, adalimumab or isotype control. The results showed that sCAR-T-induced upregulation of E-selectin, VCAM1, and ICAM1 as well as IL6, IL8, and MCP-1 by HUVEC was significantly inhibited by adalimumab (**Figures 4A–C**). In addition, the induction of TF by sCAR-T was effectively diminished by adalimumab (**Figures 4A, D**). Moreover, adalimumab effectively inhibited sCAR-T incubation-induced production of Ang2 as well as leakage of endothelial monolayer (**Figures 4E, F**).

To investigate the impact of TNFα neutralization on CAR-T cell effector function, CAR-T cells were co-cultured with Nalm6 in the presence of adalimumab or isotype control. No significant antibody-mediated toxicity was observed, suggesting that adalimumab did not affect the function of CAR-T cells *in vitro* (**Supplementary Figure 3A**).

Furthermore, we examined the effects of adalimumab on pro-inflammatory signaling pathways. The results showed that the phosphorylation of MAPK (p38, ERK, JNK) as well as the degradation of IкB were almost totally inhibited by adalimumab treatment (**Figure 4G**).

### The Synergistic Effect of TNFα and IL1β Blockade on Endothelial Activation Upon the Stimulation of Cytokine Complex Derived From Engaged CAR-T Cells and Activated Myeloid Cells

**Figure 5:**
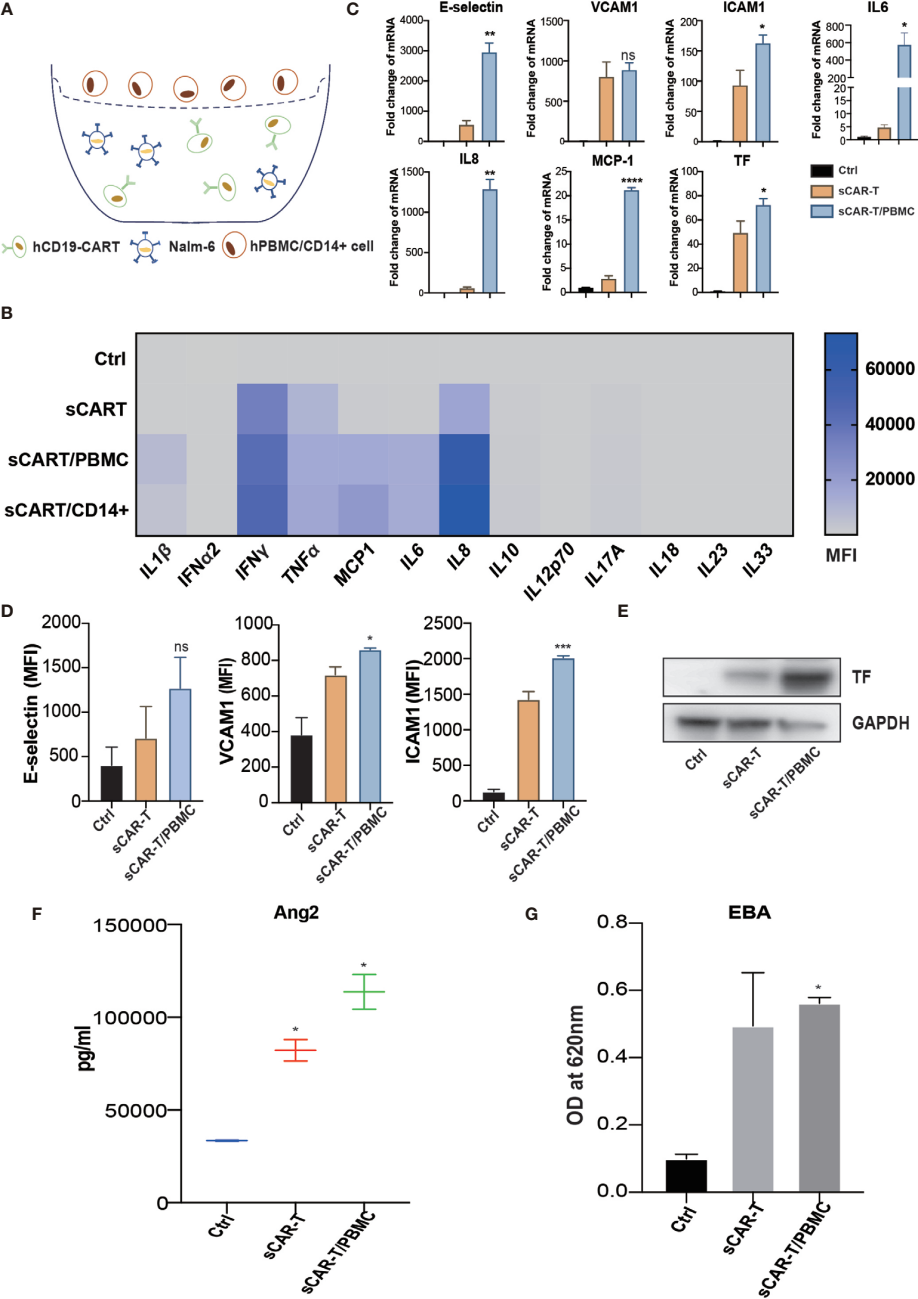
The cytokines secreted by myeloid cells enhanced sCAR-T-induced endothelial activation. **(A)** Schematic presentation of CAR-T/Nalm6/PBMC or CAR-T/Nalm6/CD14+ cells co-culture in Transwell. Details refer to the information in the Materials and Methods. **(B)** The cytokine profiles of co-cultured supernatants were determined by multi-analyte flow assay. **(C)** The mRNA levels of endothelial activation-associated markers were determined by RT-PCR. GAPDH was taken as the housekeeping gene and data was expressed as fold changes relative to control. n = 3. **(D)** The protein expression of E-selectin, VCAM1, and ICAM1 was determined by flow cytometry. n = 3. **(E)** The protein expression of TF in HUVEC was determined by western blot and GAPDH was taken as the loading control. **(F)** The concentration of Ang2 secreted by HUVEC was assessed by ELISA. n = 3. **(G)** Confluent HUVEC cultured in Transwell were incubated with sCAR-T supplemented with or without adalimumab for 12h. The permeability of endothelial monolayer was determined by EBA. * represents p < 0.05, ** represents p < 0.01, *** represents p < 0.001, and **** represents p < 0.0001. ns represents not significant. All data were representative of at least three independent experiments.

Myeloid cells are considered to be the main source of cytokines upon pro-inflammatory stimuli. To mimic the complexed cytokine condition *in vivo*, we co-cultured CAR-T cells and Nalm6 with CD14^+^ cells or PBMC (peripheral blood mononuclear cells) (**Figure 5A**). The results showed that a high level of IL1β, IFNγ, IL6, and IL8 was secreted by CD14^+^ cells and PBMC in the presence of CAR-T/Nalm6 (**Figure 5B**). The cytokine profiles were similar between PBMC and CD14^+^ cells, thereby PBMC was used for the following experiments. HUVEC were incubated with the supernatant of co-cultured CAR-T/Nalm6 (sCAR-T) and co-cultured CAR-T/Nalm6/PBMC (sCAR-T/PBMC), respectively. We found that the overall level of endothelial activation induced by sCAR-T/PBMC was significantly higher than that induced by sCAR-T (**Figures 5C–G**).

**Figure 6:**
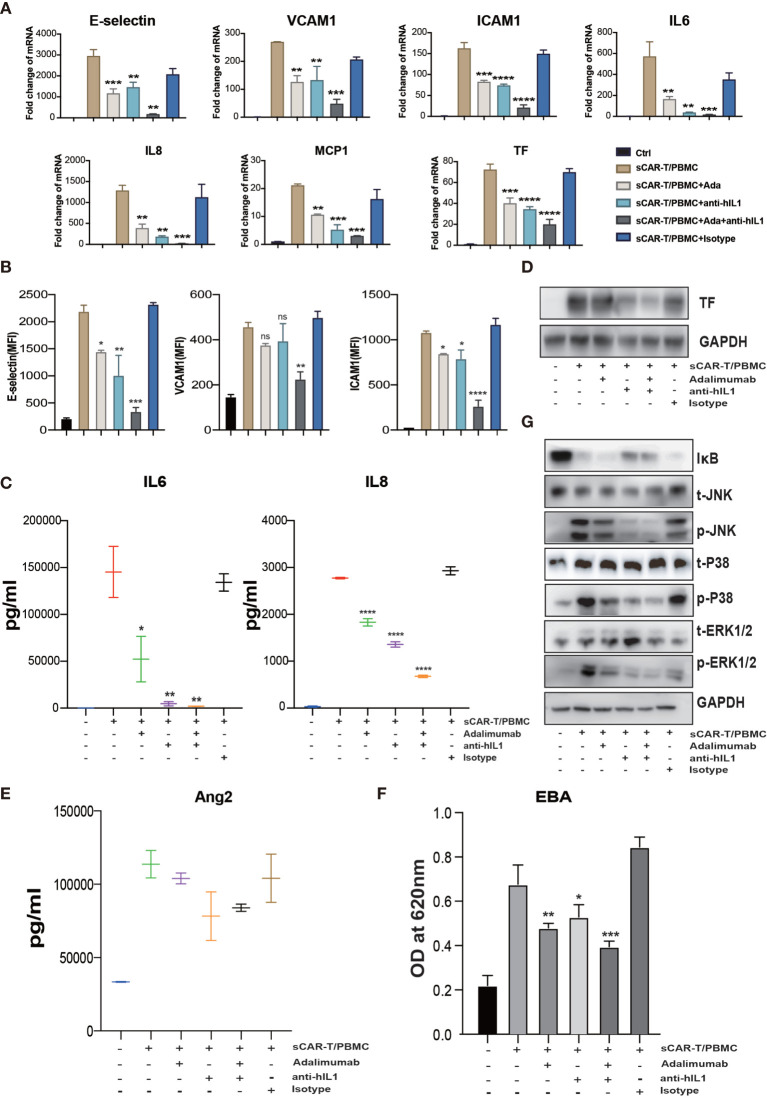
The synergistic effects of TNFα and IL1β blockade on endothelial activation induced by CAR-T/Nalm6/PBMC co-cultured supernatant. HUVEC were incubated with CAR-T/Nalm6/PBMC co-cultured supernatant supplemented with perceptively or both adalimumab (10 µg/ml) and anti-hIL1β (20 µg/ml) or isotype control for 4h. **(A)** The mRNA levels of endothelial activation-associated markers were determined by quantitative RT-PCR. GAPDH was taken as the housekeeping gene and data was expressed as fold changes relative to control. n = 3. **(B)** The protein expression of E-selectin, VCAM1, and ICAM1 was determined by flow cytometry. n = 3. **(C)** The concentration of IL6 and IL8 in the supernatant was determined by ELISA. **(D)** The protein expression of TF in HUVEC was determined by western blot. **(E)** The concentration of Ang2 secreted by HUVEC was assessed by ELISA. n = 3. **(F)** Confluent HUVEC cultured in Transwell were incubated with sCAR-T supplemented with or without adalimumab for 12h. The permeability of endothelial monolayer was determined by EBA. **(G)** The protein levels of indicated proteins were analyzed by western blot. * represents p < 0.05, ** represents p < 0.01, *** represents p < 0.001, and **** represents p < 0.0001. ns represents not significant. All data were representative of at least three independent experiments.

To evaluate the effects of cytokine released by myeloid cell mentioned above on endothelial activation, we stimulated HUVEC with human recombinant IL6/sIL6R and IL1β respectively. Our results showed that IL1β highly activated endothelial cells by upregulating the expression of adhesion molecules (E-selectin, VCAM1 and ICAM1) (**Supplementary Figure 4A**), whereas the expression of pro-inflammatory markers in endothelial cells was slightly upregulated when stimulated with IL6 and sIL6R (**Supplementary Figure 4B**), suggesting that the trans-IL6 signaling may not be the major player in CAR-T therapy-induced endothelial activation. Therefore, we speculated that IL1β is another major pro-inflammatory cytokine activating endothelial cells. By blocking TNFα and IL1β with adalimumab and anti-IL1β antibody respectively, the sCAR-T/PBMC induced-upregulation of E-selectin, VCAM1, ICAM1, IL6, IL8, MCP-1, TF, and Ang2 was inhibited to a certain level (**Figures 6A–E**). The leakage of endothelial monolayer induced by sCAR-T/PBMC was also significantly reduced by adalimumab and anti-IL1β antibody (**Figure 6F**). The combination of TNFα and IL1β blockade with adalimumab and anti-IL1β antibody showed pharmacologically synergistic effects by showing stronger inhibition of endothelial activation. In addition, IL1β blockade with anti-IL1β antibody was shown to be more effective in inhibiting the degradation of IкB as well as the phosphorylation of MAPKs induced by sCAR-T/PBMC (**Figure 6G**). Moreover, the presence of adalimumab and anti-IL1β antibody has no effect on the function of CAR-T cells *in vitro* (**Supplementary Figure 3A**).

**Figure 7:**
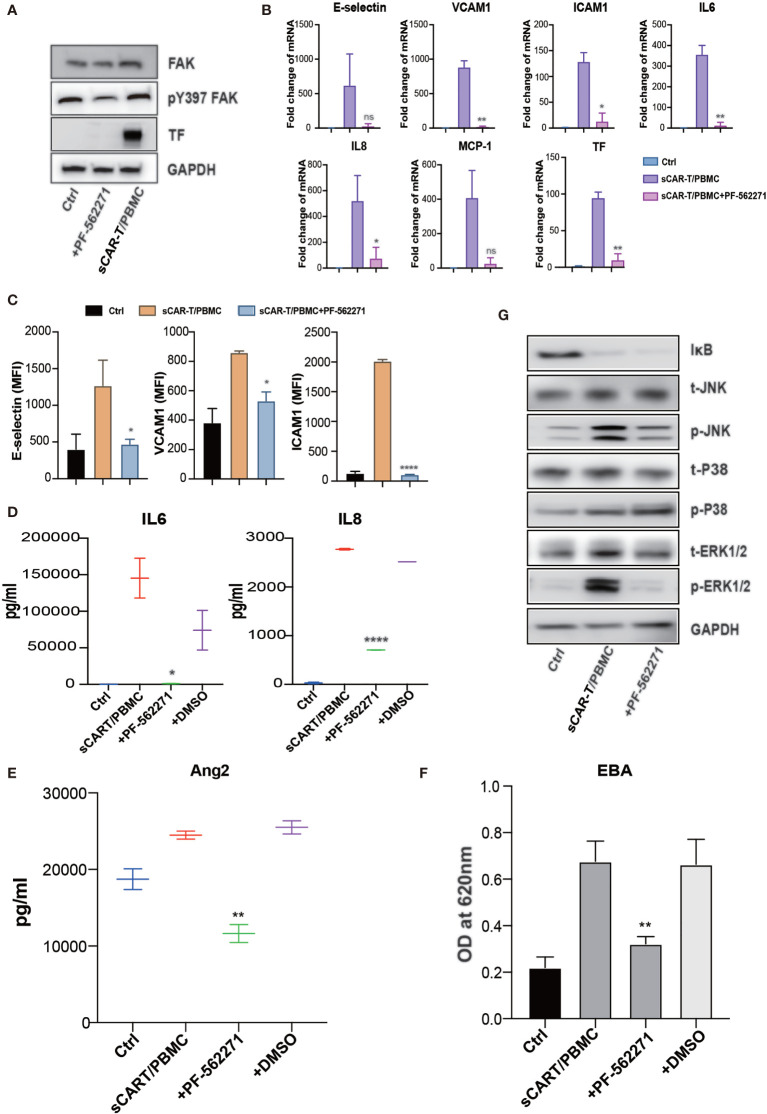
FAK inhibition effectively abolished endothelial activation induced by CAR-T/Nalm6/PBMC co-cultured supernatant. **(A)** The protein level of phosphorylated FAK at tyrosine 397 as well as TF was determined by western blot. **(B)** The mRNA levels of endothelial activation-associated markers were determined by RT-PCR. GAPDH was taken as the housekeeping gene and data was expressed as fold changes relative to control. n = 3. **(C)** The protein expression of E-selectin, VCAM1, and ICAM1 was determined by flow cytometry. n = 3. **(D)** The concentration of IL6 and IL8 in the supernatant was determined by ELISA. **(E)** The concentration of Ang2 secreted by HUVEC was assessed by ELISA. n = 3. **(F)** Confluent HUVEC cultured in Transwell were incubated with sCAR-T supplemented with or without adalimumab for 12h. The permeability of endothelial monolayer was determined by Evans blue-BSA assay. **(G)** The expression levels of indicated proteins were determined by western blot. * represents p < 0.05, ** represents p < 0.01, and **** represents p < 0.0001. ns represents not significant. All data were representative of at least three independent experiments.

Focal adhesion kinase (FAK) is a multifunctional integrin-associated protein tyrosine kinase which plays an important role in vascular development and cell migration (19, 20). FAK was reported to regulate TNFα and IL1β signaling-induced vascular endothelial adhesion molecule expression and has been demonstrated to be a therapeutic potential in the treatment of vascular inflammatory diseases and atherosclerosis (21). We found that sCAR-T/PBMC did induce FAK phosphorylation in endothelial cells and the phosphorylation level of FAK at tyrosine 397 was reduced by inhibiting the activity of FAK with specific inhibitor PF-562271 (**Figure 7A**). Endothelial activation-related markers induced by sCAR-T/PBMC were strongly inhibited by PF-562271 treatment (**Figures 7B–E**). sCAR-T/PBMC-induced endothelial leakage was also inhibited by PF-562271 (**Figure 7F**). In addition, the inhibition of FAK reduced the phosphorylation of ERK1/2 and JNK but not p38, while the degradation of IкB was not affected (**Figure 7G**). The function of CAR-T cells was not affected by PF-562271 *in vitro*, as shown in **Supplementary Figure 3B**. These data suggest that FAK might be a potential therapeutic target for the intervention of endothelial activation in CAR-T therapy.

## Discussion

CRS and ICANS are life-threatening adverse events which are detrimental obstacles to the wide application of CAR-T immunotherapy in the clinic and closely associated with the mortality of patients. Studies have shown that patients with CRS and ICANS are accompanied with strong endothelial activation-related inflammation, coagulopathy, and enhanced vascular permeability, which underline the vital roles of endothelial cells in the development of CRS and ICANS. However, the detailed mechanisms of endothelial activation in CAR-T therapy remain unclear yet. In the present study, we found that TNFα was among the most abundant cytokines secreted by engaged CAR-T cells and identified a remarkable TNFα signaling pathway activation based on the analysis of transcriptome of treated endothelial cells. In addition, IL1β released by activated myeloid cells, together with TNFα, synergistically induced endothelial activation. The blockade of TNFα and IL1β signaling with blocking antibodies as well as FAK inhibitor showed significant amelioration of endothelial activation induced by the co-culture supernatant of CAR-T/Nalm6/PBMC.

Vascular endothelium is not only crucially important for delivering oxygen and nutrients throughout the body under homeostatic condition, but also an active participant in immune responses by contributing to the initiation and perpetuation of inflammation (22). In addition, vascular endothelium plays an important role in maintaining blood brain barrier integrity. During CAR-T cell therapy, vascular endothelial cells are directly exposed to multiple stimulus in the bloodstream. The presentation of vascular inflammatory responses, capillary leakage, and consumptive coagulopathy after CAR-T therapy suggested that endothelial activation/dysfunction coincides with severe CRS/ICANS. Furthermore, it has been reported that endothelial activation and blood-brain-barrier disruption promote CAR-T-induced ICANS after adoptive immunotherapy (16, 17, 23). Severe CRS was accompanied with high serum concentration of vWF and Ang2 during endothelial activation, dysregulating endothelial coagulation function and vascular integrity (17). Moreover, Obstfeld et al. have demonstrated endothelial cells as the important source of IL6 in CRS (10), indicating the important role of activated endothelial cells in aggravating CRS/ICANS.

Cytokines produced by engaged CAR-T cells play important roles in CRS/ICANS. TNFα has been implicated to play a major role in the pathogenesis of inflammation-associated diseases like asthma, rheumatoid arthritis, and inflammatory bowel disease (24–26). High elevation of TNFα has been reported in CAR-T-treated patients, which was corelated with the significant toxicities (27). TNFα exerts its biologic effects through two receptors, TNF receptor 1 (TNFR1) and TNF receptor 2 (TNFR2), among which TNFR1 is involved in the inflammatory process and is the main TNFα receptor on endothelial membrane (28). It has been shown that TNFR1 knock down effectively inhibited TNFα-induced cytokine production in rheumatoid arthritis (29). We found that the level of engaged CAR-T-induced endothelial activation was inhibited by knocking down TNFR1 in HUVEC (**Supplementary Figure 5**), indicating that TNFR1 mediates, at least partially, endothelial activation induced by engaged CAR-T cells. In addition, the inhibition of downstream signaling pathways of TNFR1, NF-кB and MAPK, with specific small molecule inhibitors also effectively inhibited endothelial activation (**Supplementary Figure 6**). Therefore, TNFR1-NF-кB/MAPK signaling may play an important role in mediating engaged CAR-T-induced endothelial activation. However, further experiments are needed to clarify the specific mechanism. It has been reported that TNFα blockade by etanercept in combination with tocilizumab could effectively reverse CRS in some severe cases (30, 31). The inhibition effect on engaged CAR-T-induced pro-inflammatory responses, leakage, and coagulation dysfunction of endothelial cells by adalimumab suggested the benefit of TNFα blockade in maintaining endothelial function during CAR-T therapy. GM-CSF secreted by CAR-T cells has been reported to be associated with the development of grade 3 or 4 neurotoxicity in CAR-T treatment (5). Sterner et al. have demonstrated that GM-CSF neutralization with lenzilumab and GM-CSF knockout in CAR-T cells interrupted CAR-T cell-induced monocyte activation as well as subsequent IL1β and IL6 production, thereby preventing CRS and neuro-inflammation (32). This provides a viable therapeutic strategy for CAR-T therapy-related adverse events. Similarly, the strong activation of endothelial cells by CAR-T cells-derived TNFα as shown in our study indicates that knockout of TNFα in CAR-T cells might be a potential strategy for the treatment of CAR-T-induced CRS and ICANS *via* inhibiting endothelial activation.

IL1 has been reported to precede IL6 production by several hours to induce the secretion of IL6 as well as sIL6R (33). Therefore, it has been speculated that CRS might be primarily initiated by IL1 release from circulating monocytes, and IL1 blockade by anakinra was shown to be highly effective due to its systemically pharmacological intervention, including the responsiveness of neurotoxicity in mouse model (14, 15, 34). This further confirmed the vital role of IL1 in the development of CRS and ICANS during CAR-T therapy. We showed that the combined blockade of TNFα and IL1β with adalimumab and anti-IL1β antibody could effectively reduce endothelial activation induced by sCAR-T/PBMC, which might be the potential strategy to ameliorate and prevent the syndrome of CART-associated toxicities.

Lethal neurotoxicity in CAR-T therapy has emphasized the importance of better understanding of this severe pathology. Patients with severe neurotoxicity demonstrated the evidence of blood brain barrier disruption which leads to the leakage of high concentrations of systemic cytokines into the cerebrospinal fluid (17). Blood brain barrier is made up of endothelial cells with tight junction. Ang1/Ang2-Tie2 axis plays a critical role in modulating endothelial permeability. It has been reported that the level of Ang2 concentration at early stage (day 0 and day 1) after CAR-T infusion corelated with the severity of subsequent neurotoxicity, indicating that endothelial activation occurs early after CAR-T therapy and precedes the onset of neurotoxicity (17). In our study, CAR-T/Nalm6/PBMC co-culture-induced Ang2 secretion by activated endothelial cells tend to be inhibited by TNFα and IL1β blockade as well as FAK inhibition, implying the potentially protective effects of these interventions on CAR-T-associated neurotoxicity. It needs to be noted that the decrease of Ang2 was not statistically significant. The presence of both CAR-T-derived and myeloid cells-derived cytokines makes the cytokine profiles more complicated. The blockade of TNFα and IL1β signaling may lead to the compensatory effect of other pro-inflammatory cytokines. For instance, the levels of IFNγ and IL8 were significantly higher in the supernatant of CAR-T/Nalm6/PBMC in our study. IFNγ has been demonstrated to induce endothelial injury. Harijith et al. have reported that Ang2 induced by IFNγ may affect caspase-dependent cell death pathways and contribute to alveolar simplification (35). Moreover, the synergistic effects of different cytokines cannot be neglected. For instance, it has been reported that TNFα and IFNγ synergistically induced a lethal cytokine shock in a mouse model of COVID-19 (36). Therefore, the induction of Ang2 by other cytokines may explain the insignificant decrease of Ang2 by the blockade of TNFα and IL1β signaling in our result. This suggest us that a compensatory mechanism may overcome the blockade of TNFα and IL1β secreted by CAR-T and PBMC, thus promoting HUVEC activation and possible CRS/ICANS events *in vivo*. The pharmacological effects of TNFα/IL1β neutralizing antibodies as well as FAK inhibitor on CAR-T therapy-induced endothelial dysfunction still need to be verified *in vivo*, and the optimization of the interfering strategies might also be needed.

This is the first report providing evidence on vascular endothelial activation by CAR-T cells and myeloid cells-derived cytokine complex. Our study reveals that patients may benefit from the prevention of endothelial activation during CAR-T therapy. The effects of TNFα and IL1β blocking antibodies as well as FAK inhibitor on CAR-T therapy-induced endothelial dysfunction need to be verified *in vivo*. Notably, it has been reported that currently available preclinical models are poorly predictive of the clinical behavior of CAR-T cells due to the lack of bystander human hematopoiesis, which makes it difficult to investigate CAR-T-mediated toxicities with these models. Recently, Norelli et al. have reported a humanized murine model recapitulating all major toxicities induced by CD19 CAR-T cells in human, therefore allowing the study of CRS and neurotoxicity *in vivo* (14). Therefore, we are trying to perform our *in vivo* experiments using humanized mice to reproduce CAR-T-associated CRS and neurotoxicity *in vivo* and to verify our present findings in the future studies.

## Conclusions

**Figure 8:**
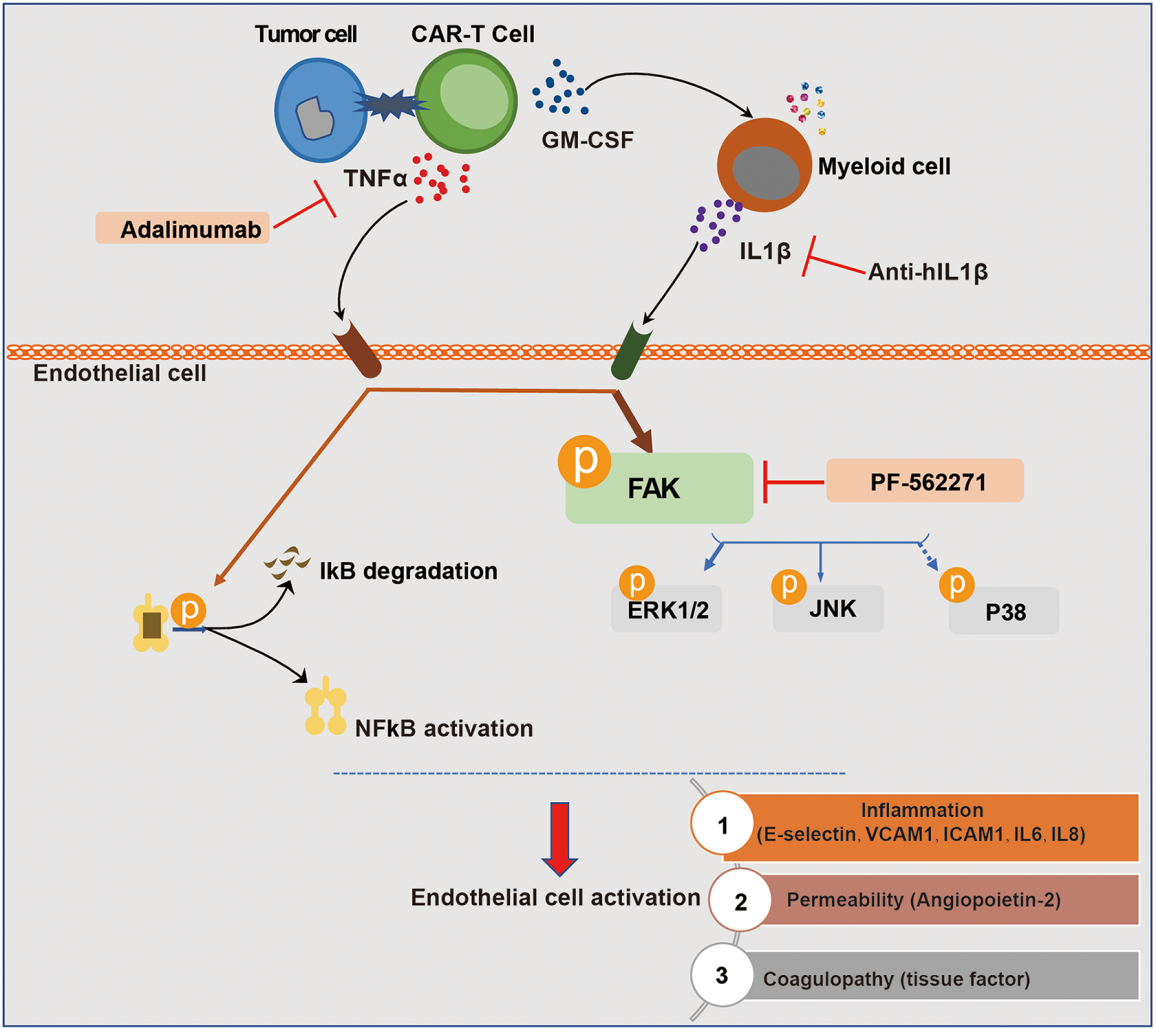
Schematic description of the role of endothelial activation in CAR-T therapy-induced CRS and neurotoxicity. The recognition of tumor cells by CAR-T cells leads to the activation of CAR-T cells and the production of cytokines. Among the most abundant cytokines secreted by engaged CAR-T cells, TNFα played a major role in inducing endothelial activation. CAR-T cells and tumor cells induced the activation of myeloid cells, leading to the secretion of IL1β which was another important pro-inflammatory cytokine that induces endothelial activation. In this process, FAK, NF-кB, and MAPK were activated. The blockade of TNFα and IL1β with adalimumab and anti-IL1β as well as the inhibition of FAK activity effectively ameliorated endothelial dysfunction induced by CAR-T/tumor cells/myeloid cells in CAR-T therapy.

In conclusion, our study demonstrated that among the cytokines produced by engaged CAR-T cells per se and myeloid cells activated by CAR-T therapy, TNFα and IL1β were the main mediators of vascular endothelial activation, which are potential targets of cytokine therapy for CAR-T-associated CRS and neurotoxicity. In addition, the inhibition of FAK, the critical kinase mediating both TNFα and IL1β signaling, effectively ameliorated endothelial dysfunction induced by CAR-T/tumor cells/myeloid cells (**Figure 8**). Our study revealed that by interfering with vascular endothelial activation, it might be potent to alleviate CAR-T therapy-induced CRS and neurotoxicity and to improve the safety of CAR-T immunotherapy, for which the clinical benefits still needs to be further investigated.

## Data Availability Statement

The datasets presented in this study can be found in online repositories. The names of the repository/repositories and accession number(s) can be found below: NODE (The National Omics Data Encyclopedia),project NO. OEP001728 (https://www.biosino.org/node/project/detail/OEP001728).

## Author Contributions

YC and RL performed experiments, analyzed data, and wrote the manuscript. SS and XY assisted with experiments. YW, WW, and LL supervised the study and wrote the manuscript. All authors contributed to the article and approved the submitted version.

## Funding

This work was supported by National Natural Science Foundation of China (81770182), Shanghai Municipal Education Commission-Gaofeng Clinical Medicine Grant (20152507), and Shanghai Jiao Tong University Tang Scholar Program (2017).

## Conflict of Interest

The authors declare that the research was conducted in the absence of any commercial or financial relationships that could be construed as a potential conflict of interest.
