# Supplementary material for: Therapeutic Potential of TNFα and IL1β Blockade for CRS/ICANS in CAR-T Therapy via Ameliorating Endothelial Activation
Source: Front Immunol. 2021 May 19;12:623610. doi: 10.3389/fimmu.2021.623610 (PMC8170323; doi:10.3389/fimmu.2021.623610)
Supplement: Supplementary file 1 [file DataSheet_1.docx]

**Supplemental materials**

**Methods**

**Generation of hCD19-CAR lentivirus**

Lentivirus was prepared according to previous publications(1). Briefly, when the 293T cells in T75 flask were at 80~90% confluent, add mixture of target-DNA plasmid containing CAR sequence(1)/packing plasmid and jet PRIME® (Polyplus NO.114-15) at a ratio of 1:2 according to the protocol of manufacturer. Harvest the viral medium after 48 to 72h. Filter the viral supernatant through a 0.45µm filter. Then ultracentrifuge retroviral supernatant using 40ml Ultra-Clear Centrifuge Tubes (BECKMAN COULTER, NO.344058) at 28000 g, 4°C for 2h. After concentrating the lentivirus particles, measure the titer as described previously. Split the virus particles and store at -80°C for further experiments.

**CAR-T cell manufacture**

Thawed primary human peripheral blood mononuclear cells (PBMC) were cultured in PRIME-XV T cell CDM (91154, Irvine SCIENTIFIC), supplemented with 100IU/ml human recombination IL2 (13238-050, ThermoFisher). Human PBMC were activated using Dynabeads human T-expander CD3/CD28 (00775135, Gibco) at a beads: cell ratio of 3:1. Coat non-tissue culture plates with RetroNectin (T100A, Takara) at 20 μg/ml according to the protocol of the manufacture ahead of transfection. Human activated PBMCs were transfected at a multiplicity of infection (MOI) of 10~30 supplemented with HitransG A 25X (REVG004, GENE). Centrifuge cell/virus mixture at 1000 g, at 32°C for 90min. Incubate at 37°C with 5% CO_2_ for another 48h. Analyze transduction efficiency of T cell by FACS after 2 to 3 days. Count T cells every other day and maintain a T cell concentration of 1 to 2 × 10^6^ cells/mL in T25 flask for optimal expansion and viability.

**Cytotoxicity assay**

For the cytotoxicity assays, the GFP-Luciferase^+^ Nalm6 or the GFP-Luciferase^-^ cells were incubated with or without CAR-T cells at E:T ratio of 2:1 for 6h. Samples were treated with 1 µl D-luciferin (30 µg/ml) per 100 µl sample volume prior to measurement. The killing capacity of CAR-T cells was calculated by measuring the absorbance for the residual live tumor cells using Varioskan Flash (Thermofisher).

**Small interfering RNA-mediated gene silencing**

TNFR1 was knocked down using small interfering RNA (siRNA) sequence for TNFR1. Negative control siRNA was used as control. TNFR1 siRNA and negative control siRNA were purchased from GenenPharma (Shanghai, China). HiPerFect (301704, QIAGEN, Germany) was used as the transfection reagent according to the manufacturer's instructions.

1. Milone MC, Fish JD, Carpenito C, Carroll RG, Binder GK, Teachey D, et al. Chimeric receptors containing CD137 signal transduction domains mediate enhanced survival of T cells and increased antileukemic efficacy in vivo. *Mol Ther* (2009) 17(8):1453-64. doi: 10.1038/mt.2009.83.

**Supplementary Figure 1. The validation of main cytokines released by endothelial cells activated by engaged CAR-T cells at different T:E ratios.** The concentration of IL6 and IL8 in the supernatant of endothelial cells activated by engaged CAR-T cells at different T:E ratios was determined by ELISA. * represents p < 0.05, *** represents p < 0.001. ns represents not significant. All data were representative of at least three independent experiments.

**Supplementary Figure 2.** **Verification of key genes involved in endothelial activation by quantitative RT-PCR.** The mRNA levels of selected key genes were analyzed by quantitative RT-PCR. GAPDH was taken as the housekeeping gene and data was expressed as fold changes relative to control n = 3. * represents p < 0.05. All data were representative of at least three independent experiments.

**Supplementary Figure 3. The effect of blocking agents on the function of hCD19 CAR-T cells. (A)** GFP-Luciferase^+^ Nalm6 or the GFP-Luciferase^-^ cells were incubated with or without CAR-T cells at E:T ratio of 2:1 in the presence of adalimumab/anti-IL1 antibody for 6h. The absorbance of the residual live tumor cells was measured to calculate the killing capacity of CAR-T cells. **(B)** GFP-Luciferase^+^ Nalm6 or the GFP-Luciferase^-^ cells were incubated with or without CAR-T cells at E:T ratio of 2:1 in the presence of PF-562271 for 6h. The absorbance of the residual live tumor cells was measured to calculate the killing capacity of CAR-T cells. ns represents not significant. All data were representative of at least three independent experiments.

**Supplementary Figure 4.** **The effect of IL1β, IL6/sIL6R combination on endothelial activation. (A).** HUVEC were stimulated with IL1β (10ng/ul) for 4h, and the protein expression of E-selectin, VCAM1, and ICAM1 was determined by flow cytometry. n = 3 (**B).** HUVEC were stimulated with IL6/sIL6R for 4h, and the protein expression of E-selectin, VCAM1, and ICAM1 was determined by flow cytometry. n = 3.

**Supplementary Figure 5.** **The effects of TNFR1 knock down on sCAR-T-induced endothelial activation.** HUVEC were transfected with negative control siRNA or TNFR1 siRNA for 48h, and then incubated with sCAR-T for another 4h. (**A)**. The mRNA expression of endothelial activation-related genes and TNFR1 was determined by quantitative RT-PCR. GAPDH was taken as the housekeeping gene and data was expressed as fold changes relative to control n = 3. (**B)**. The protein levels of TNFR1, TF, E-selectin, VCAM1, and ICAM1 were determined by western blot. * represents p < 0.05. ns represents not significant. All data were representative of at least three independent experiments, sCAR-T in siRNA NC group vs. sCAR-T in siRNA TNFR1 group.

**Supplementary Figure 6.** **The effect of small molecule inhibitors of NF-𝜅B and MAPKs on sCAR-T-induced endothelial activation.** HUVEC were incubated with sCAR-T supplemented with NF-𝜅B inhibitor BAY11-7082, JNK inhibitor SP600125, ERK1/2 inhibitor S7524, and p38 inhibitor SB203580 respectively. **(A).** The mRNA expression of endothelial activation-related genes was determined by quantitative RT-PCR. GAPDH was taken as the housekeeping gene and data was expressed as fold changes relative to control. n = 3. (**B)**. The protein levels of indicated proteins were determined by western blot. (**C)**. The expression levels of E-selectin, VCAM1, and ICAM1 were determined by flow cytometry. n = 3. * represents p < 0.05, ** represents p < 0.01, *** represents p < 0.001, and **** represents p < 0.0001. ns represents not significant. All data were representative of at least three independent experiments.

**
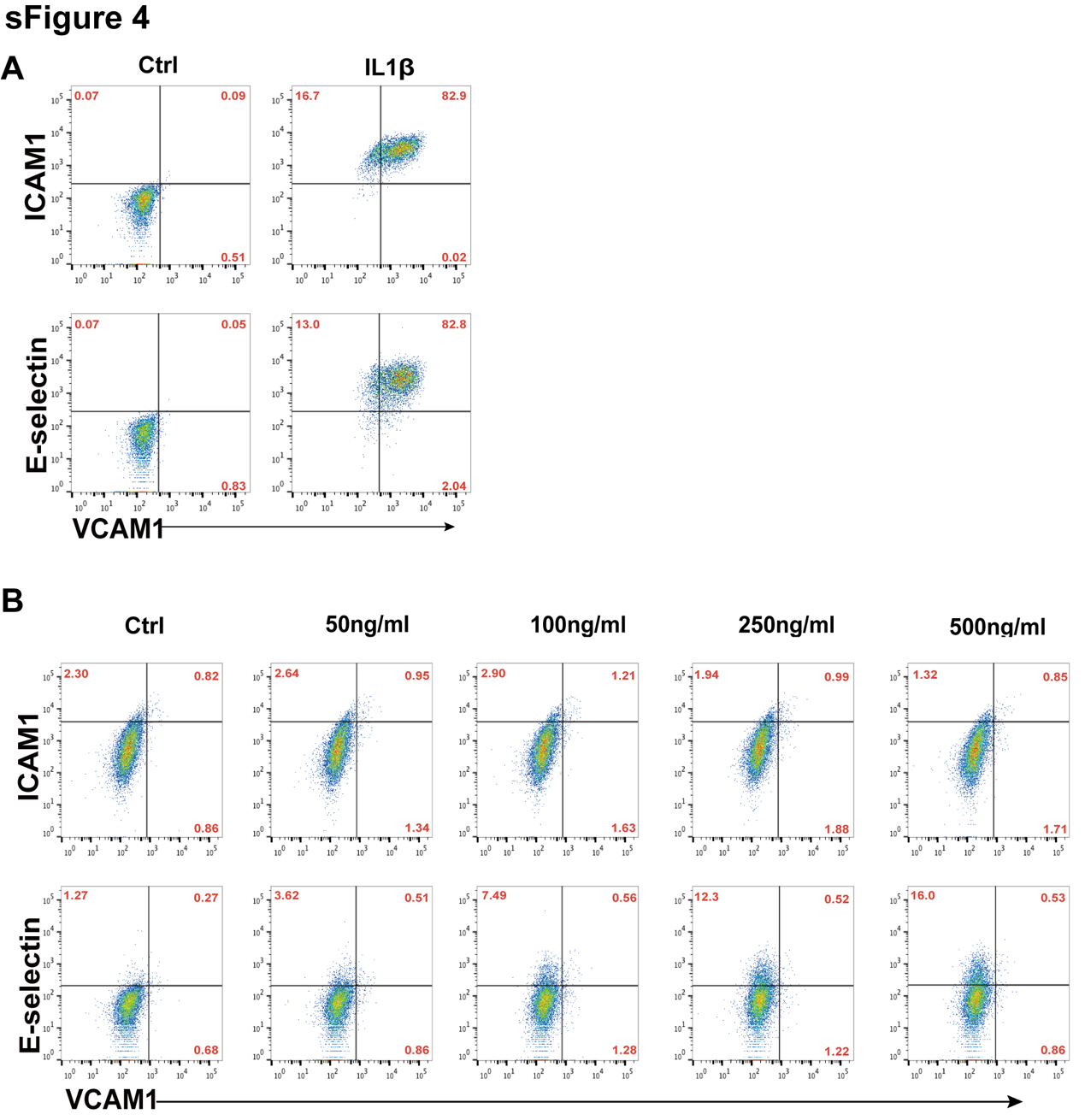
**

**
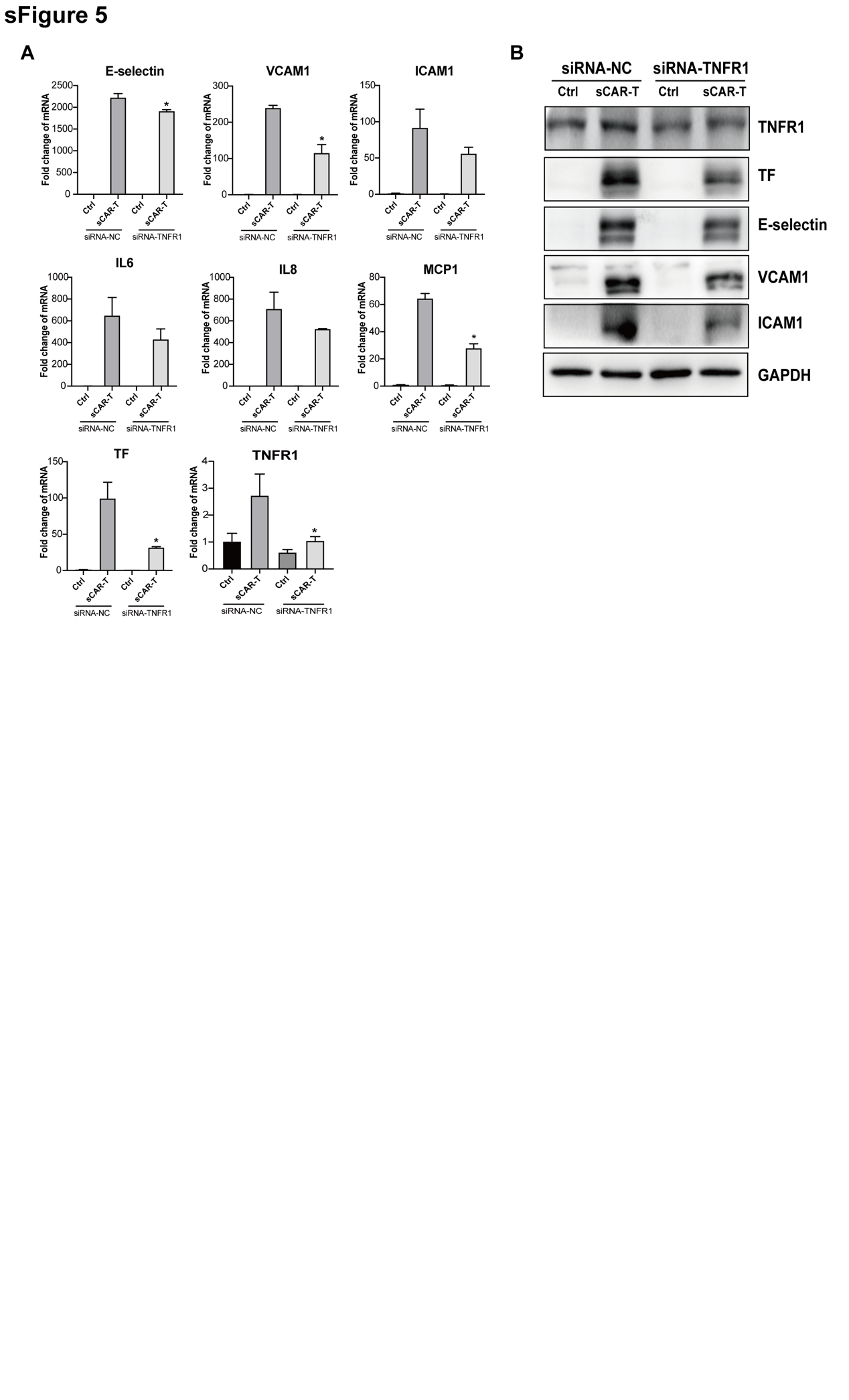
**

**
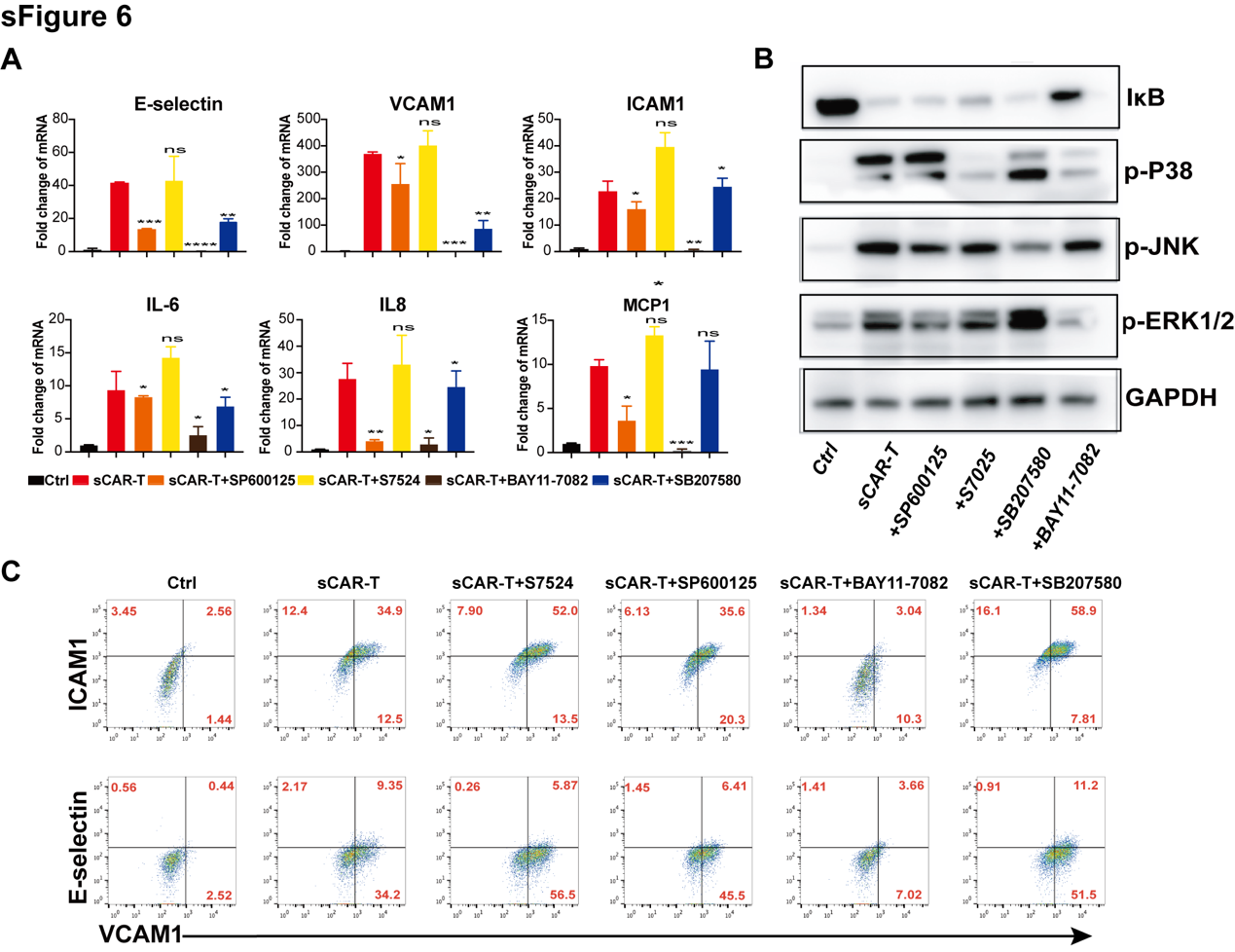
**

**Supplementary Table 1. Sequences of human primers for quantitative RT-PCR.**

| **Gene name** | **Forward** | **Reverse** |
| --- | --- | --- |
| E-selectin | 5′‐GAAGAGGTTCCTTCCTGCCAAGTG‐3′ | 5′‐CAGAGCCATTGAGCGTCCATCC‐3′ |
| VCAM1 | 5′ACCACATCTACGCTGACAATGAATCC‐3′ | 5′‐AACACTTGACTGTGATCGGCTTCC‐3′ |
| ICAM1 | 5′‐GTCACCTATGGCAACGACTCCTTC‐3′ | 5′‐AGTGTCTCCTGGCTCTGGTTCC‐3′ |
| IL6 | 5′‐GCAAAGAGGCACTGGCAGAA‐3′ | 5′‐TGCACAGCTCTGGCTTGTTC‐3′ |
| IL8 | 5′‐TGCAGCTCTGTGTGAAGGTG‐3′ | 5′‐TGCACAGCTCTGGCTTGTTC‐3′ |
| MCP1 | 5’-CCCCAGTCACCTGCTGTTAT-3′ | 5’-CAGATCTCCTTGGCCACAAT-3′ |
| TF | 5′ACGAGATTGTGAAGGATGTGAAGCAG‐3′ | 5′TGTCTCCAGGTAAGGTGTGAACTCTG‐3′ |

**Supplementary Table 2. The absolute levels of different cytokines and factors released by sCAR-T-stimulated endothelial cell.**

**T:E ratio**

| **Analyte** | **1:0**  **(pg/ml)** | **1:1**  **(pg/ml)** | **1:2**  **(pg/ml)** | **1:10**  **(pg/ml)** |
| --- | --- | --- | --- | --- |
| **TNFα** | 83.2 | 117.3 | 700.0 | 1309.0 |
| **IL-6** | 39.5 | 785.8 | 4149.5 | 5109.0 |
| **vWF-A2** | 392.2 | 362.8 | 469.8 | 581.7 |
| **IL-8** | 389.9 | 3766.0 | 12890.0 | 16215.5 |
| **IL-10** | 12.7 | 14.2 | 23.7 | 24.6 |
| **MCP-1** | 994.6 | 4315.5 | 6665.0 | 8692.5 |
| **IL-6 Rα** | 95.1 | 88.0 | 100.7 | 107.8 |
| **IL-1β** | 65.5 | 61.5 | 74.0 | 79.5 |
| **IFNγ** | 365.6 | 1640.0 | 9440.0 | 15939.5 |
| **IL-1Rα** | 1321.5 | 1290.5 | 1624.0 | 1754.0 |
| **PAI-1** | 52074 | 46830.5 | 52023.0 | 54785.5 |
| **GM-CSF** | 49.0 | 710.3 | 6232.0 | 11827.5 |
| **CD25** | 109.9 | 163.3 | 408.8 | 506.2 |
| **VEGF-A** | 59.9 | 137.0 | 298.2 | 416.9 |
| **IL-15** | 35.3 | 39.0 | 50.915 | 53.6 |

**Supplementary Table 3. The absolute levels of different cytokines and factors in sCAR-T.**

**T:E ratio**

| **Analyte** | **1:0**  **(pg/ml)** | **1:1**  **(pg/ml)** | **1:2**  **(pg/ml)** | **1:10**  **(pg/ml)** |
| --- | --- | --- | --- | --- |
| **TNFα** | 9.0 | 41.9 | 589.0 | 1343.0 |
| **IL-6** | 3.8 | 3.8 | 5.1 | 6.4 |
| **vWF-A2** | 45.6 | 48.9 | 63.3 | 97.7 |
| **IL-8** | 6.9 | 573.4 | 2688.0 | 4722.0 |
| **IL-10** | 7.9 | 8.4 | 11.4 | 14.6 |
| **MCP-1** | 66.9 | 72.7 | 98.3 | 142.3 |
| **IL-6 Rα** | 39.9 | 50.6 | 45.2 | 56.1 |
| **IL-1β** | 21.0 | 20.0 | 21.0 | 23.0 |
| **IFNγ** | 70.3 | 1223.0 | 7811.0 | 16623.0 |
| **IL-1Rα** | 25.3 | 59.4 | 130.3 | 208.3 |
| **PAI-1** | 46.2 | 154.4 | 258.4 | 425.2 |
| **GM-CSF** | 14.2 | 582.9 | 5246.0 | 12057.0 |
| **CD25** | 52.8 | 150.9 | 318.4 | 448.8 |
| **VEGF-A** | 15.0 | 27.4 | 148.7 | 275.8 |
| **IL-15** | 6.3 | 7.4 | 8.7 | 12.7 |
